# “You Always Have It in the Back of Your Mind”—Feelings, Coping, and Support Needs of Women with Pathogenic Variants in Moderate-Risk Genes for Hereditary Breast Cancer Attending Genetic Counseling in Germany: A Qualitative Interview Study

**DOI:** 10.3390/ijerph19063525

**Published:** 2022-03-16

**Authors:** Claudia Stracke, Clarissa Lemmen, Kerstin Rhiem, Rita Schmutzler, Sibylle Kautz-Freimuth, Stephanie Stock

**Affiliations:** 1 Institute of Health Economics and Clinical Epidemiology, Faculty of Medicine and University Hospital Cologne, University of Cologne, 50935 Cologne, Germany; clarissa.lemmen@uk-koeln.de (C.L.); sibylle.kautz-freimuth@uk-koeln.de (S.K.-F.); stephanie.stock@uk-koeln.de (S.S.); 2 Center for Familial Breast and Ovarian Cancer, Center for Integrated Oncology (CIO), Faculty of Medicine and University Hospital Cologne, University of Cologne, 50937 Cologne, Germany; kerstin.rhiem@uk-koeln.de (K.R.); rita.schmutzler@uk-koeln.de (R.S.)

**Keywords:** genetic counseling, hereditary breast cancer, moderate-risk breast cancer genes, coping, qualitative research, support needs

## Abstract

Hereditary breast cancer accounts for approximately 30% of newly diagnosed breast cancer (BC) cases. Pathogenic variants in moderate-risk BC genes (MBCG) differ from those in high-risk genes in terms of associated cancer risks, affected organs, and available preventive options. Little is known about how MBCG pathogenic variant carriers who have attended post-test genetic counseling perceive their situation, how they cope with their situation, and which support needs they might have. Problem-centered, guided, individual interviews were conducted with twelve women carrying pathogenic variants in MBCG. The interview analysis was based on Mayring’s qualitative content analysis. The women were between 29 and 59 years old and carried pathogenic variants in the risk genes *CHEK2* (n = 8), *ATM* (n = 1), or *PALB2* (n = 3). Women reported a wide range of feelings, both positive (relief, calmness) and negative (overwhelm, fear, grief, guilt). All women applied strategies of emotion-focused coping to deal with this lifelong situation. Appraisal and evaluation of the affected mother’s coping might influence the patient’s own behavior and coping style. These results could be used during and after post-test genetic counseling to provide more needs-oriented counseling, and to help women in adjusting to and coping with being a pathogenic variant carrier.

## 1. Introduction

Hereditary breast cancer accounts for 30% of newly diagnosed breast cancer (BC) cases and is linked with germline pathogenic variants in the high-risk genes *BRCA1* or *BRCA2* in approximately 24% of cases [1,2]. These pathogenic variants are associated with a lifelong risk of approximately 70% for BC and approximately 17% (*BRCA1*) to 44% (*BRCA2*) for ovarian cancer (OC) [3]. Advances in next-generation sequencing and multigene panels have enabled the identification of additional moderate-risk BC genes (MBCG) that also play a role in the development of hereditary BC, such as *CHEK2*, *ATM*, *RAD51C/D*, and *PALB2*. Two recent case–control studies confirmed with very large cohorts that there is a significant association between pathogenic variants in these genes and the development of BC [4,5]. Current research indicates that *CHEK2*, *RAD51C/D*, and *ATM* pathogenic variants are associated with a lifetime risk of BC of approximately 20% to 35% and are accordingly regarded as moderate-risk genes [4,5,6,7,8]. *PALB2* is associated with a lifetime risk of BC of approximately 50% and rather considered to be a moderate-to high-risk gene [9]. In addition, male *PALB2* pathogenic variant carriers also have an increased risk of BC [9]. Further cancer risks that are associated with these MBCG are pancreatic cancer for *ATM*, *CHECK2*, and *PALB2*, and colon and prostate cancer for *CHEK2* [10,11,12,13]. *RAD51C/D* pathogenic variants are associated with an increased risk for OC, and recent data on *PALB2* indicate an increase in the risk of OC as well [9,12,14,15].

With the expanding use of gene-panel testing, counseling women with pathogenic variants in MBCG will become increasingly important for the clinical setting [16]. Given this scenario, the question arises of how well medical providers are equipped to advise these patients [16]—not only regarding the above-mentioned cancer spectrum and risk estimates, but also regarding preventive options that can be offered to the carriers of these pathogenic variants. In Germany, women carrying MBCG pathogenic variants are generally recommended to follow the multimodal intensified breast surveillance program for the breast (physical examination, sonogram, MRI) from the age of 30 [17]. These preventive options are explained to the women in person by a specialized doctor at the post-test genetic counseling (PTGC). They also receive a brochure containing information on the multimodal intensified breast surveillance program. For women with pathogenic variants in the high-risk genes *BRCA1/2,* additional preventive options are offered: besides this multimodal intensified breast surveillance program, they can opt for a risk-reducing surgery of the healthy breast tissue and/or the adnexa. According to current recommendations in Germany, these risk-reducing surgeries are usually not recommended for women with pathogenic variants in MBCG and they usually only have the choice to participate in the multimodal intensified breast surveillance program [18,19]. Tung et al. [20] emphasize that a threshold risk that justifies risk-reducing mastectomy (RRM) has not been established, yet. Narod [16] also points out that “preventive salpingo-oophorectomy is not justified, and preventive mastectomy is a questionable approach for women with a lifetime breast cancer risk of 20 to 25%. For the majority of women with a mutation in *ATM* or *CHEK2*, management consists of screening alone”. Since the associated cancer risks are smaller but the uncertainty is higher, women with pathogenic variants in MBCG face a different and especially challenging situation compared to carriers of pathogenic variants in the high-risk genes. Genetic counselors must therefore be prepared to communicate less certain risks and less evidenced management options to women carrying pathogenic variants in MBCG [21]. In order to address this particular situation in the counseling session, not only in terms of medical information needs but also on an emotional level, it is important and helpful to have information about women’s feelings, coping mechanisms, and support needs. According to Waltz et al. [21], research on how to communicate risk and treatment decisions concerning BC genes beyond *BRCA1/2* is crucial. Furthermore, studies focusing on high-risk pathogenic variants should not be extrapolated to those who carry moderate-risk pathogenic variants [20,22]. Reyes et al. conducted a qualitative study with women carrying a *CHEK2* or *ATM* pathogenic variant and focused on this very special experience of uncertainty regarding specific cancer risk estimates and regarding the effectiveness of certain risk management strategies [22]. Beyond this, to the authors’ knowledge, emerging feelings, coping styles, and support needs of women confronted with a pathogenic variant in a MBCG have not been assessed so far. The aim of this study was to obtain a closer insight into (1) how the women perceive the situation following the disclosure of the genetic test result, (2) the personal coping strategies of women carrying pathogenic variants in MBCG, (3) the influence of the familial medical history on the coping style, and (4) what kind of additional support the women need to cope with their life-long increased cancer risk.

## 2. Materials and Methods

To obtain a first overview, an initial systematic literature search on the situation of women with pathogenic variants in MBCG was performed in five databases (Pubmed, CINAHL, EMBASE, PsychINFO, Cochrane). Most of the studies that were identified focused on the situation of *BRCA1/2* pathogenic variant carriers or of women who were at high risk of hereditary BC but had not been tested. Hence, a qualitative approach was chosen to gain a deeper insight into the situation of women with pathogenic variants in MBCG, using in-depth, semi-structured, individual, face-to-face interviews. This research was supported by the European Commission for the HORIZON 2020 project BRIDGES (Breast Cancer Risk after Diagnostic Gene Sequencing), grant number 634935. The conduct and analysis of the study were based on the COREQ (Consolidated Criteria for Reporting Qualitative Studies) Checklist, a checklist of 32 items that should be included in reports of qualitative research [23]. The TRAPD (Translation, Review, Adjudication, Pretesting, and Documentation) EES translation guidelines [24] were applied to translate the quotes used in this manuscript from German into English. This method included two translators, who produced parallel independent translations of the German quotes, and one further person to review and compare the different versions and discuss them with the translators. The preferred translations were selected through consensus.

### 2.1. Post-Test Genetic Counseling

In Germany, genetic counseling is mandatory before genetic testing, as declared by the German Genetic Diagnostics Act [25]. Women who test positive for a pathogenic variant that is associated with an increased risk of BC are then invited to a personal PTGC, where they are informed in detail about their test result, associated cancer risks, and their preventive options. The PTGC takes place in a specialized center for familial breast and ovarian cancer and is carried out by a doctor who is a specialist in gynecology and obstetrics with specialized training in hereditary breast and ovarian cancer or a specialist in human genetics. All the participants in this study attended a personal PTGC at the Center for Familial Breast and Ovarian Cancer at the University Hospital of Cologne. During this counseling session, the women received detailed, individual, oral information regarding their test results, the probability of them inheriting the pathogenic variant, associated cancer risks, and preventive measures that could be offered to the women. With regard to potentially associated cancer risks other than BC and OC (e.g., pancreatic cancer), further consultation in a specialized center is recommended to the women. Additionally, women receive the following written information: a brochure on hereditary breast and ovarian cancer in general, a brochure on the multimodal intensified breast surveillance program, a doctor’s letter that summarizes the PTGC and offers information on psycho-oncological support, and an information letter addressed to their relatives. Further information about the PTGC can be found in Stracke et al. [26].

### 2.2. Participants and Recruitment

The participants were recruited using purposive sampling [27]. Inclusion criteria covered women with pathogenic variants in one of the risk genes *CHEK2*, *RAD51C*, *RAD51D*, *PALB2*, or *ATM*, either with or without a personal history of BC and/or OC, and who were at least 18 years old and in a medically and psychologically stable condition. To ensure this stable condition, recruitment was carried out by experienced gynecologists during a medical consultation, as this situation allowed them to assess the women’s medical and psychological states. Further inclusion criteria were an adequate understanding of the German language and a time interval to the PTGC of at least 6 months to ensure a temporal and emotional distance from the disclosure of the genetic test result. Eligible women were identified by the Center for Familial Breast and Ovarian Cancer at the University Hospital of Cologne and invited to participate in the study. Women interested in participating in the study were contacted to obtain consent, and a face-to-face interview was arranged. Since no *RAD51C/D* pathogenic variant carrier met the inclusion criteria during the recruitment period, it was not possible to include women with this pathogenic variant in the study. Data collection depended on the criterion of theoretical saturation and the recruitment process was closed after the 12th interview, since it was possible to assign more than 80% of the 85 codings derived from this interview to pre-existing codes and no additional data or themes appeared [28,29].

### 2.3. Data Collection

In-depth, semi-structured, individual, face-to-face interviews were carried out between February 2017 and October 2017. Interviews were conducted by the first and fifth authors; both were female and had a medical background. To avoid social desirability bias, none of the interviewers were involved in the PTGC. To ensure familiarity with the counseling situation, both interviewers observed several PTGCs at the Center for Familial Breast and Ovarian Cancer prior to the start of the interviews.

The interview guide was based on the problem-centered interview (PCI) [30]. This method was chosen because the PCI focuses on the interviewees’ experiences and perceptions of a very specific topic, in this case being a carrier of a pathogenic variant in an MBCG. The interview guide was developed based on theoretical assumptions about the perceptions, problems, and experiences of women with a risk of hereditary BC derived from the findings of the initial systematic literature search. The interview guide began by asking about the reasons and circumstances that motivated or influenced the woman to undergo genetic testing. This pre-formulated, open introductory question was supposed to center the conversation on the problem under study. Further questions to generate storytelling and comprehension were applied during the course of the interview and provided a communicative atmosphere. Categories and topics to be addressed during the interview and explored through various guiding questions were, for example, the perception and atmosphere of the PTGC, information that was unclear/not understood, feelings during the PTGC, communication with the family after the PTGC. One pretest interview was conducted with one woman with a *CHEK2* pathogenic variant to pilot the interview guide. This pretest interview was not included in the analysis. The interview guide was further revised as new topics emerged during the course of the interviews. Additional instruments of the PCI included the short questionnaire to collect data on social and medical characteristics, and the postscript to document situational and nonverbal aspects. The interviews were conducted at the Center for Familial Breast and Ovarian Cancer, the Institute of Health Economics and Clinical Epidemiology, the BRCA-Network in Bonn, or the participant’s hometown and lasted 75 to 180 min. All the interviews were audiotaped and transcribed verbatim [31]. Approval was obtained from the Ethic Commission of the Medical Faculty of the University of Cologne (ethic votes of 22 November 2016, reference number 16-098).

### 2.4. Data Analysis

Mayring’s qualitative content analysis was applied to systematically analyze the interview transcripts, using a category system [32]. The category system consists of main categories and subcategories. The initial main categories (= codes) were developed based on the interview guide and the theoretical assumptions derived from the findings of the initial systematic literature search. Initial main codes included, for example, “Opinion about personal psycho-oncological counseling” and “Opinion about self-help groups”. During the coding process, further relevant themes and categories emerging from the data were identified and categorized by the first author into main categories and subcategories. These categories, which emerged from the data, included, for example, the categories “Feelings associated with being a carrier of a pathogenic variant” and “Impact of family medical history.” Codes and categories were validated internally with the second and the fifth authors after the fourth interview. After the last interview, the first, second, and fifth authors discussed and reviewed the final codebook until a consensus was reached. The MAXQDA software program was used to assist in coding and extracting the data. Additionally, the qualitative results were quantified, and percentages provided to give an impression of the frequency of the studied topics.

## 3. Results

### 3.1. Study Participants

Twelve women participated in this study. The percentages mentioned below always refer to the total number of 12 women. Table 1 gives an overview of the characteristics of the study participants. Participants were between 29 and 58 years old and the majority had children (92%). Eight women (67%) had a positive pathogenic variant status for the *CHEK2* gene, three (25%) for the *PALB2* gene, and one (8%) for the *ATM* gene. Nine women (75%) were affected by BC in the past (referred to as survivors in the following) and three women (25%) had no history of BC (referred to as previvors in the following). The majority (92%) had a positive family history for BC in the nuclear family (mother or sister) or extended family (aunt, cousin, grandmother). Educational level was classified into “No formal education” (8%), “Vocational training” (67%), and “Bachelor’s degree or above” (25%). To enable an international comparison, corresponding ISCED 2011 (International Standard Classification of Education) levels have been added [33].

### 3.2. Results of Qualitative Analysis

The thematic analysis of the interview data produced 815 codings that were categorized into 81 codes. The codes were organized into main categories and subcategories and further systematized to identify key areas and major themes. Six major themes were identified, showing results on (1) feelings associated with the disclosure of the genetic test result, (2) feelings associated with the overall situation, (3) feelings associated with being a carrier of a pathogenic variant, (4) coping styles, (5) impact of familial medical history, and (6) support needs.

Table 2 gives an overview of these major themes and subthemes that emerged from the data and the representative quotes. The order of the subthemes is according to the number of women. Each of the major themes is discussed in detail below.

#### 3.2.1. Feelings Associated with the Disclosure of the Genetic Test Result

At the time of the disclosure of the genetic test result during the PTGC, eight women (67%), both survivors and previvors, reported that they had felt overwhelmed by the situation. One of them remembered:

*“I have not been receptive at this point of time” [47 years old, survivor, CHEK2].*

Another woman stated:

*“Yes, although I had expected the genetic test result to be positive, I have to say I was a bit shocked when I received this information. And then you are, I guess, not that receptive” [38 years old, survivor, ATM].*

At the same time, six out of the twelve participants (50%), both survivors and previvors, also experienced feelings of relief at the time of the disclosure of the genetic test result. Four of them (33%) experienced both relief and overwhelm. One of them describes this confluence:

*“On the one hand, the relief. Yes, it is something. You can explain now why it was like that with my mother. But of course, there is also this shock. Oh my God, now you have it too. What will happen, if?” [38 years old, previvor, CHEK2].*

The different reasons for the participants feeling relief are described in Table 3.

Three women felt relief because—against their expectation—their test result was negative for *BRCA*1/2 pathogenic variants. One participant felt relief because she finally had an explanation and confirmation of her increased risk. One previvor was very relieved not to have *BRCA1/2* and because she finally had something concrete to hold onto. One woman was relieved because, with her having a pathogenic variant, her daughter and sister were included into the multimodal intensified breast surveillance program of the university hospital.

The three participants (25%) who did not feel overwhelmed reported feelings of certainty and indifference regarding the moment of the genetic test disclosure. They all had experienced women suffering from BC in their nuclear family. One participant said:

*“But you think to yourself, ‘Yes…well…okay…you were expecting it. Just go home. Have a cup of coffee (laughing)’” [29 years old, previvor, PALB2].*

Further feelings arose during the PTGC. Three previvors experienced grief through the memory of deceased loved ones at the moment of genetic test disclosure:

*“So, at the first moment, I immediately had to think of my sister because she had that, too” [34 years old, previvor, CHEK2].*

Two women, both survivors, explained that the genetic test disclosure was not as stressful and upsetting as the previous BC diagnosis had been:

*“That means this whole thing, the breast cancer diagnosis the year before, that was a lot more upsetting and awful” [50 years old, survivor, CHEK2].*

One participant was not accompanied by friends or family and felt lonely during disclosure.

#### 3.2.2. Feelings Associated with the Overall Situation

Concerning the overall situation, seven women (58%) experienced feelings of fear or concern (Table 4). These feelings have been described in the context of (1) the health of children or blood relatives, (2) developing BC, (3) the BC therapy and its sequelae, (4) abnormal findings in the intensified breast surveillance program or a recurrence, and (5) unclear statements and information concerning the risks associated with the pathogenic variant.

On the other hand, five participants (42%), all survivors, expressed rather feelings of calmness and acceptance towards the overall situation. One participant stated:

*“I’ll now tell you quite honestly. In my case, there is nothing I can do about it now” [47 years old, survivor, CHEK2].*

Another woman shared this view:

*“I do everything that can be done. I regularly go for checkups” [55 years old, survivor, CHEK2].*

Furthermore, four women (33%), two survivors and two previvors, experienced a feeling of a lack of understanding by their relatives regarding their way of coping with the pathogenic variant. *“They [the children] have blocked this” [47 years old, survivor, CHEK2],* one participant said. Another woman stated: *“They [the relatives] could not understand this” [38 years, previvor, CHEK2].* Four women (33%) expressed a high need for safety, which was expressed in the fact that they would be willing to do anything (including risk-reducing surgery) to minimize their cancer risk in the future. One woman explained:

*“And I say very clearly, if there is something, operate straight away, full stop” [55 years old, survivor, CHEK2].*

For three women (25%), all survivors with a family history of BC, it was important to distance themselves from the feelings and needs of other women who were in similar situations. One explained right at the beginning of the interview that she might not be the right person to talk to because she was different from others due to her individual medical family history. Another woman shared this view:

*“Then I’ll just say what I consider important, so that maybe afterwards you can understand why I might have very different needs than any of the other women you’re interviewing” [58 years old, survivor, PALB2].*

#### 3.2.3. Feelings Associated with Being a Carrier of a Pathogenic Variant

Concerning the feelings associated with being a carrier of a pathogenic variant, one participant spoke about *“the burden of passing the gene on to the children” [50 years old, survivor, CHEK2]* and the fear associated with it. She also pointed out:

*“It’s also about not having any resentment towards your parents, and I think that’s very, very important, especially with such a disease that is inherited” [50 years old, survivor, CHEK2].*

Another participant talked about her father’s feelings about being a carrier of a pathogenic variant:

*“Well, he [the participant’s father] doesn’t say it like that, he always says he didn’t have that much time. But I do think so. Yes, I think he also has a bit of a guilty conscience” [44 years old, survivor, CHEK2].*

Furthermore, the women’s feelings might also be influenced by the behavior or statements of (female) relatives. One woman describes the repelling behavior of her niece after the genetic test disclosure:

*“Well, for example, my niece/I have a problem there now. We got along great with each other (...). Therefore, I was so disappointed and hurt. That was really hard” [50 years old, survivor, CHEK2].*

#### 3.2.4. Coping

Women with pathogenic variants for hereditary BC must cope with this situation their whole life. One aim of this study was to find out how these women manage to cope with their pathogenic variant and the lifelong uncertainty and to identify resources that they draw on. Coping was defined and classified according to the Transactional Model of Stress and Coping by Lazarus and Folkmann [34] into emotion-focused coping, problem-focused coping, and meaning-focused coping. While emotion-focused coping refers to the thoughts and actions people use to regulate or reduce distress, problem-focused coaching refers to strategies people use to manage or solve the problem that is causing the distress. Meaning-focused coping helps the person to make sense of what is happening and appraise benefit where possible. Table 5 gives an overview of the different types of coping and strategies that were identified and are presented in the following.

All twelve women mentioned resources or coping strategies assigned to emotion-focused coping. A very important aspect was the support from their partner, family, and friends, which was mentioned by eight participants (67%). Another very important aspect mentioned by six women (50%) was strategies that can be summarized as self-care strategies. This includes meditation, mindfulness, and consideration of one’s own needs and desires and not only those of others. Further emotion-focused coping strategies were psychotherapy and self-help groups, sports and nature-related activities, religion, and acceptance and repression.

Ten women (83%) described strategies classified as problem-focused coping. Taking part in the multimodal intensified breast surveillance program of the university hospital was mentioned by nine out of twelve women (75%). Another important aspect was health-promoting behavior such as healthy eating or exercise. Health-promoting strategies were mentioned by four (33%) participants. In addition, for two women (17%), the direct confrontation with the pathogenic variant and the disease was important to cope with their situation. Further problem-focused coping strategies included willingness to undergo (risk-reducing) surgery and becoming more aware of their own health and more sensitive towards body changes.

Five women (42%), all survivors, mentioned aspects that reframe or reappraise the situation in positive ways and that can be classified as meaning-based coping. Three (25%) were happy to be able to share the knowledge and experience that they gained due to their BC disease with their children and other women; one of them even considered writing a book about her experiences. Two women stated that, because of their own BC disease, they became aware of what is good for themselves, and that one should enjoy life to the fullest and be grateful. Two became aware of their own personal strengths in the way they handled the overall situation. Another woman explained that it was only through psycho-oncology counseling during her BC disease that she was able to address pre-existing underlying psychological conditions that she did not deal with before.

#### 3.2.5. Impact of Familial Medical History

Except for one woman, all participants had a family history of BC, either in their nuclear (67%) or extended (25%) family. This experience might have influenced the women’s feeling, views, and coping style. Three women (25%) whose mothers died of cancer mentioned that they had already dealt with the topics of disease and death very intensively:

*“For me, it [death] is just part of it because my parents passed away very early. For me, it’s just an ordinary topic” [49 years old, survivor, PALB2].*

Another aspect of the family medical history was the evaluation and appraisal of the patient’s mother’s way of dealing with her own diagnosis of BC. Three women expressed a negative opinion about their mothers’ way of dealing with their cancer disease. One woman explained:

*“This generation doesn’t go to see a doctor. This is a generation that closes its eyes and sticks its head in the sand. (…) That’s why I deal with those things completely differently than my mother. And I am simply a completely different person in such matters” [55 years old, survivor, CHEK2].*

On the other hand, one woman expressed a positive opinion about how her mother dealt with the BC disease:

*“Well, I am not afraid at all of actually getting sick. Not at all. My mom did a good job” [29 years old, previvor, PALB2].*

#### 3.2.6. Support Needs

Five women (42%) indicated that they did not need either personal psycho-oncological counseling or support through self-help groups. For two of them, one survivor and one previvor, and both with a medical family history of BC, talking to family members was more important than talking to counselors or therapists. One of them said:

*“It was offered to me, to seek psychological help. But I did not want that for myself. Well, I am someone, I say of myself, I can handle that quite well because I have seen it until the end with my mother. (…) Family is more important to me, before I talk to someone else about it” [38 years old, previvor, CHEK2].*

The other one had a very close relationship with her sister, who also carried a MBCG pathogenic variant and had been affected by BC, and with whom she talked a lot about the pathogenic variant and how to deal with the BC disease. The other three women did not mention an intensive exchange with their family but had no need for psycho-oncological counseling or support that was offered through a self-help group, either.

Five out of twelve women (42%) mentioned that they had already seen a therapist or counselor. Two of them, both survivors, shared very positive experiences about it:

*“Also with the cancer, we also talked about that. So, there were many things, that had to be sorted out. And that helped me quite well” [47 years old, survivor, CHEK2].*

This participant even stated that she would still be interested in further specialized counseling as offered by the university hospital, but that it was too far away from her hometown to make use of this offer. The other woman revealed:

*“And I perceived that [the personal psycho-oncological counseling] really helpful during that time, again and again and again” [58 years old, survivor, PALB2].*

Both were not interested in self-help groups. The other three women attended counseling once in the past but reported that they had no need for personal psycho-oncological support regarding the pathogenic variant. Two of them, one survivor and one previvor, were interested in self-help groups instead. One of them was a previvor without any contact with other pathogenic variant carriers in the family and the other woman was a survivor, who described very positive experiences regarding self-help groups during her BC disease:

*“Well, after my experience with psychologists, that’s quite nice, but I believe that if you talk to other affected people, you can, yes, much more relate to that. You have much more empathy and you get told much more what you can do, right?” [50 years old, survivor, CHEK2].*

Two interviewees indicated that they would be interested in both psycho-oncological support and self-help groups. One was a survivor and the other one was a previvor, and both experienced the disease through their mothers and had close relationships with their families.

Eventually, some interviewees added general comments regarding the topic of psycho-oncological counseling. Three of them considered it important to have local services and that there was no need to travel to the university hospital. One woman emphasized that it was important for the psycho-oncology counselor to have medical professional knowledge.

## 4. Discussion

The aim of this study was to explore the feelings, coping styles, and support needs of women confronted with a pathogenic variant in a MBCG. After conducting an initial systematic literature search in five databases (Pubmed, CINAHL, EMBASE, PsychINFO, Cochrane), a qualitative approach was chosen to gain first insights into the feelings and coping situations of these women.

The results of the study revealed a wide range of feelings, both positive and negative. Overwhelm and being emotionally and/or mentally overloaded at the moment of the disclosure of the genetic test result were mentioned by almost all participants, regardless of whether they were survivors or previvors. Studies have shown that feelings of overwhelm and emotional overload may arise when severe diseases are diagnosed. Samson et al. [35] indicate that the disclosure of the genetic test result to unaffected *BRCA1/2* pathogenic variant carriers can be perceived to be as threatening as being diagnosed with BC. Augestadt et al. [36] conducted a qualitative study with women newly diagnosed with BC or OC who were consecutively offered testing for *BRCA1* and *BRCA2* pathogenic variants at the time of cancer diagnosis without receiving genetic counselling prior to genetic testing. Many of these women already felt overwhelmed and mentally overloaded when being offered genetic testing because of the amount of information that they received. The results of our study indicate that not only the diagnosis of a severe disease and the offer of genetic testing can be overwhelming and stressful, but also the disclosure of the genetic test result. This is in line with Leonarczyk and Mawn [37], who conducted a qualitative study with *BRCA1/2* previvors. The participants also experienced the diagnosis of the *BRCA1/2* pathogenic variant as profoundly traumatic and overwhelming. The participants of our study also described that—due to this overwhelm—they were not able to fully assimilate the genetic and medical information provided orally during the PTGC. According to Kessels [38] and Brown et al. [39], recall of medical information is low in general, suggesting that 80% of medical information provided by healthcare professionals is forgotten. In addition, genomic literacy in the general population is very low [39,40] and confusion of genetic terms is particularly common [41]. There is a clear need to provide women with additional written information media, either as a written information booklet or web-based in the form of a website or app, to make sure that they comprehend what the pathogenic variant implies for their health and that of their families.

A further feeling associated with the disclosure of the genetic test result that was identified was relief that—other than expected—the genetic test result was negative for *BRCA1/2* pathogenic variants. Since *BRCA1/2* pathogenic variants are associated with a higher cancer risk, it may comfort some women that the test result is negative. This finding is in line with Reyes et al. [22], who recently studied the uncertainty experienced by carriers of *ATM* and/or *CHEK2* pathogenic variants. Participants of this study also reported relief due to the relatively lower risk of developing BC compared to individuals with pathogenic variants in high-risk genes, such as *BRCA1/2*. This information might be comforting and helpful for MBCG pathogenic variant carriers and may be communicated during the PTGC. However, Waltz et al. [21] examined the use of *BRCA1/2* as a reference or gold standard in communicating other BC-related genes and its possible implications for patients. They found out that the use of *BRCA1/2* as “anchors” may confuse patients and impact their understanding of the uncertainty. Hence, this should be paid attention to when using *BRCA1/2* as a reference at the PTGC. Both feelings, overwhelm and relief, can occur at the same time and demonstrate the complexity of feelings that can occur and with which the genetic counselor may be confronted during the PTGC.

Some feelings arose only or mainly in different subgroups. Some survivors expressed feelings of calmness or acceptance while previvors expressed fear and concern, especially when they had already experienced the BC disease through their mother. Grief and sadness were experienced during disclosure by those who had already lost close family members because of BC. This variety of feelings indicates that the feelings of affected women may differ from those of unaffected women and that the family medical history might have an influence on their needs and feelings as well. As a consequence, information and counseling might need to be adapted to these specific situations to make sure that all feelings and needs are appropriately addressed.

Concerning the feelings associated with being a carrier of a pathogenic variant, several feelings came up during the interviews: feelings of guilt, the burden of inheriting a pathogenic variant, the parents’ bad conscience, and the blaming of the female relatives were described by the participants. This is in line with other studies focusing on *BRCA1/2* pathogenic variant carriers. In a study by Lynch et al. [42], participants revealed feelings of guilt about passing the pathogenic variant on to their children and worry about their children developing cancer. The authors identified these concerns as two of the four major burdens of carriers of pathogenic variants. Hallowell [43] and Grant et al. [44] use the term “genetic responsibility” to describe this special situation and the feeling of being responsible for the health of others. Fisher et al. [45] focused on the offspring’s perspectives and described how the daughter expressed blame towards her mother for having passed along her increased risk. These findings indicate that in order to prevent blame and negative feelings, there is a strong need for understandable, clear, and basic information regarding the modes of inheritance and the inheritance patterns of pathogenic variants. It might be important to explain that these inheritance patterns cannot be influenced and that there is no one to blame for anything.

All participants of this study used coping strategies that can be classified as emotion-focused coping according to the definition of Lazarus and Folkman [34], e.g., seeking support from family and friends or self-care strategies. According to Folkman, emotion-focused coping strategies refer to the thoughts and actions people use to regulate or reduce distress and are used more in situations that have to be accepted and cannot be controlled, such as chronic illness [46]. Since carrying a pathogenic variant is also something that one must accept and cannot change, the result of our study is in line with this assumption. Having an increased risk for hereditary BC implies being confronted with and challenged by uncertainty for a lifetime [22,47,48]. Studies even suggest that women who carry a pathogenic variant in an MBCG experience higher levels of uncertainty compared to carriers of a pathogenic variant in the high-risk genes *BRCA1/2* [20,22,49]. This difference might be caused by the limited empirical data about cancer risk estimates and about the effectiveness of risk management strategies for MBCG. According to Uncertainty Management Theory [50], uncertainty may not only be anxiety-inducing but also enhance coping and the aim should therefore be to manage uncertainty, not to reduce it. The coping styles of women at increased risk for hereditary BC have also been assessed by Pieterse et al. [51] in a quantitative study applying the coping styles of the Utrecht Coping List [52]. They found out that “Seeking social support” was a favorable coping style since it was associated with lower levels of psychological distress. Holland and Holahan [53] determined that a high level of social support led to higher levels of emotional well-being in BC patients, and in a study by Ozdemit and Arslan [54], social support predicted effective stress management in women with BC.

In addition to emotion-focused coping, Lazarus and Folkman define problem-focused coping as the strategies that people use to manage or solve the problem that is causing the distress [34]. The most important strategy that can be assigned to this coping style and that was mentioned by 75% of the study participants was participation in the multimodal intensified breast surveillance program of the university hospital. However, there are also qualitative studies with *BRCA1/2* pathogenic variant carriers indicating that participation in a multimodal intensified breast surveillance program might lead to fear and worry caused by (false positive) abnormal findings, follow-up tests, and biopsies. Werner-Lin [55] and Hoskins and Greene [56] use the terms “surveillance fatigue” and “screening fatigue” to describe this very special situation of young women at high risk for hereditary BC who regularly participate in intensified breast surveillance and experience the ongoing screening as stressful, uncomfortable, and scary. In these studies, surveillance fatigue was one of the reasons for young previvors choosing an RRM at an early age. According to Metcalfe et al. [57], in unaffected women who carry a *BRCA1/2* pathogenic variant, cancer-related distress decreased significantly after having both RRM and risk-reducing salpingo-oophorectomy. The participants of our study mostly experienced the multimodal intensified breast surveillance program as helpful and positive. One reason for this difference might be the age of our participants, with only one woman under the age of 30. This young previvor’s concerns were further elaborated in Stracke et al. [26] and were similar to those of the young previvors described by Werner-Lin and Hoskins and Greene [55,56]. Another reason might be the absence of an alternative prevention option other than the multimodal intensified breast surveillance program, since RRM is not generally recommended for carriers of pathogenic variants in MBCG in Germany [18,58].

A further coping style that was mentioned by 42% of the participants—all survivors—was meaning-focused coping. As with emotion-focused coping, meaning-focused coping appears to be used more in situations that are chronic and cannot be changed, such as chronic illness, and it might be used when initial coping efforts fail [46]. Studies with cancer patients found that meaning-based coping led to a restoration of well-being and was associated with a higher quality of life [59,60]. Furthermore, Vehling et al. [61] found that a global sense of meaning is an important protective factor concerning the development of distress in cancer patients.

The results of our study indicate that a family history of BC—especially in the nuclear family—might also have an impact on the coping style and the attitudes and views of the participants. Some women expressed a positive or negative opinion about how their mother dealt with the BC disease. This appraisal and evaluation of the mother’s coping might influence the patient’s own behavior and coping style. Studies have found that a family history of BC influences BC prevention decisions among healthy women at elevated risk of BC [62,63]. Furthermore, women who have a first-degree relative who is a BC survivor might be more optimistic in their beliefs about the benefits of early detection [62]. Mæland et al. [64] found that the loss of a mother due to OC affects women living with a hereditary cancer risk and influences how they deal with their genetic cancer risk.

The participants had very diverse and heterogeneous views on the topics of psycho-oncological support and self-help. While some were interested in both and expressed a relevant need, others were rather reserved, preferring their family as a primary contact. This diversity was also found in a qualitative study on the support needs of affected and unaffected *BRCA1/2* pathogenic variant carriers by Hughes and Phelps [65], indicating that “different people want different things at different stages in their life and throughout their genetic journey”. They proposed a Model of Support that consists of multiple elements, such as social events, a 24 h phone line, a chat forum, and a central organizing body, which is professional and peer-led [65]. Farrely et al. [66] suggested the implementation of a telephone-based peer support intervention for women who carry a *BRCA1/2* pathogenic variant. In addition to the calls, they encouraged the use of text messaging and/or email and concluded that a mixed-medium intervention might be preferable and the most effective. Segal et al. [67] investigated the interest in interventions to support the disclosure process of *BRCA1/2* pathogenic variant carriers to their offspring and also suggested that one type of support may not be suitable for all women. Eventually, a quantitative study on the support needs of unaffected women with a family history of BC found that demographic variables did not predict the interest in attending a support group [68].

There are a few limitations of our study. We interviewed only one woman without children, and only one woman without a family history of BC. We recommend further studies to gain deeper insights into the feelings, coping styles, and support needs of these women. Furthermore, quantitative studies are needed to explore potential differences between the different subgroups and between the different MBCG pathogenic variants. A further limitation is the selection bias. As purposive sampling was employed, the participants might have already been interested in sharing their personal experiences. Another potential limitation is the sample size, which included just twelve participants. However, a theoretical saturation was postulated since it was possible to assign more than 80% of the codings from the 12th interview to pre-existing codes. Furthermore, the results of this study may not be transferable across different settings or to institutions with other counseling standards and processes.

One of the strengths of our study is the qualitative approach, which enabled us to explore a wide range of feelings, living contexts, and circumstances on a one-to-one basis. Thanks to the protected settings of the individual interviews, the participants were able to open up and share very private thoughts and experiences and the interviewer was able to react spontaneously to statements and emotions. Another strength is the heterogeneity of the study sample: the women interviewed had pathogenic variants in different MBCG, represented both survivors and previvors, and some had completed their family planning while others had not. This provided a first in-depth insight into a broad spectrum of different perspectives, life situations, feelings, and opinions from this specific target group.

## 5. Conclusions

The aim of this study was to obtain a closer insight into the situation of women carrying pathogenic variants in MBCG. We were able to explore feelings during and following the disclosure of the genetic test result, women’s personal coping strategies, possible influences of familial medical history, and the women’s attitudes towards psycho-oncological support and self-help. This study demonstrates that women with pathogenic variants in MBCG experience a wide range of feelings at the time of the disclosure of the genetic test result during the PTGC and continue to have a wide range of feelings several months or years after the disclosure. Negative feelings such as overwhelm, fear and concern, grief, and guilt, as well as positive feelings such as relief, calmness, and acceptance, might occur, even at the same time. This should be taken into account and might be addressed by counselors and physicians at the PTGC or at the annual multimodal intensified breast surveillance appointments, if necessary. To deal with this lifelong situation and the lifelong uncertainty posed by the pathogenic variant, all participants mentioned resources or coping strategies that can be assigned to emotion-focused coping, and most participants also described strategies that are classified as problem-focused coping. Some affected women mentioned aspects that can be classified as meaning-based coping. Women with a family history of BC—especially in the nuclear family—might express a positive or negative opinion about how their mothers dealt with the BC disease. This appraisal and evaluation of their mother’s coping might influence their own behavior and coping style. These valuable insights into the women’s feelings and coping mechanisms might help to improve the PTGC sessions, to make counselling more patient-centered, and to provide more needs-oriented information for carriers of pathogenic variants in MBCG. Concerning the topics of psycho-oncological support and self-help, the participants had a very diverse and heterogeneous view. Hence, psychological support and the possibility to join self-help groups should be offered to women carrying pathogenic variants in MBCG at the PTGC as well as at follow-up appointments.

## Figures and Tables

**Table 1:** Participant characteristics (n = 12).

Characteristic of the Participants	n (%)
Age	
<30 years	1 (8%)
30–40 years	3 (25%)
41–50 years	4 (33%)
>50 years	4 (33%)
Pathogenic variant	
*ATM*	1 (8%)
*CHEK2*	8 (67%)
*PALB2*	3 (25%)
Time between PTGC and interview	
6–12 months	2 (17%)
12–36 months	5 (42%)
>36 months	5 (42%)
BC Status	
Survivor	9 (75%)
Previvor	3 (25%)
Marital status	
Married	9 (75%)
Divorced	2 (17%)
In a relationship	1 (8%)
Family history of BC	
No family history of BC (participant is index patient)	1 (8%)
BC in nuclear family	8 (67%)
BC in extended family	3 (25%)
Children	
No children	1 (8%)
One or more children	11 (92%)
Educational level/ISDEC level	
No formal education	1 (8%)
Vocational training/ISCED 3	6 (50%)
Vocational training (health sector)/ISCED 4	2 (17%)
Bachelor’s degree or above/ISCED 6 or 7	3 (25%)

PTGC = post-test genetic counseling; BC = breast cancer; survivor = pathogenic variant carrier affected by BC; previvor = pathogenic variant carrier unaffected by BC; nuclear family = parents and siblings; extended family = grandparents, cousins, aunt/uncle; vocational training = dual training system in Germany, requirement: either Secondary Education or Higher Education Entrance Qualification; ISCED = International Standard Classification of Education 2011 [33]. Percentages shown in the table have been rounded to the nearest whole percent.

**Table 2:** Major themes that emerged from the data and representative quotes.

Major Theme and Subthemes	Number of Women n (%)	Representative Quote
1. Feelings associated with the disclosure of the genetic test result		
1.1. Overwhelm	8 (67%)	*“I think I just switched off after that [the disclosure of the genetic test result] because I didn’t want to hear any more. (…) I just wanted to go home.” [34 years old, previvor, CHEK2]*
1.2. Relief	6 (50%)	*“Because then I finally had something to hold on to. So for me, there were so many people among us who died of it, and no one can tell you why. (...) Yes, it is something. You can explain it now why it was like this for my mother.” [38 years old, previvor, CHEK2]*
1.3. Certainty and indifference	3 (25%)	*“But you think to yourself, ‘Yes…well…okay…you were expecting it. Just go home. Have a cup of coffee (laughing).’” [29 years old, previvor, PALB2]*
1.4. Grief through the memory of deceased loved ones	3 (25%)	*“So, at the first moment, I immediately had to think of my sister because she had that, too.” [34 years old, previvor, CHEK2]*
1.5. BC diagnosis was perceived as worse than disclosure of pathogenic variant	2 (17%)	*“So therefore, this genetic test result was actually, I think, not that important for us anymore, I would say. (...) That [the BC diagnosis 1 year before] was rather the shock in the family.” [55 years old, survivor, CHEK2]*
1.6. Loneliness	1 (8%)	*“But it wasn’t until the doctor told me that straight to the point, I had tears running down my face. That was somehow quite strange, but yes, I would not do it again today, drive there on my own. But I didn’t really know what was going to happen because I’m not actually afraid. But this confrontation has triggered everything in me.” [47 years old, survivor, CHEK2]*
2. Feelings associated with the overall situation		
2.1. Fear and concern	7 (58%)	*“My mother became ill for the first time at the age of 40. I’m now approaching 40, and of course with every examination more and more panic comes along.” [38 years old, previvor, CHEK2]*
2.2. Calmness and acceptance	5 (42%)	*“But because I have already completed my [breast cancer] therapy, and I assume that I have lowered my risk so far. I was not worried or anxious now.” [38 years old, survivor, ATM]*
2.3. Feeling of a lack of understanding by the relatives	4 (33%)	*“And the fact that this is something that I’m concerned about. He [the husband] just can’t understand.” [49 years old, survivor, PALB2]*
2.4. Need for safety	4 (33%)	*“For me, personally, I would like to minimize all risk factors as soon as I personally can to prevent a new outbreak.” [49 years old, survivor, PALB2]*
2.5. Differentiation from the needs of other women	3 (25%)	*“Then I’ll just say what I consider important, so that maybe afterwards you can understand why I might have very different needs than any of the other women you’re interviewing.” [58 years old, survivor, PALB2]*
3. Feelings associated with being a carrier of a pathogenic variant		
3.1. Resentment	1 (8%)	*“It’s also about not having any resentment towards your parents, and I think that’s very, very important, especially with such a disease that is inherited.” [50 years old, survivor, CHEK2]*
3.2. Burden	1 (8%)	*“This burden, you can’t help it, but still this burden of passing the gene on to the children and the fear, especially if the girl maybe wants to get pregnant now and have children and, and, and, and, the hormone balance shifts. I think I would have been a bit afraid.” [50 years old, survivor, CHEK2]*
3.3. Guilt	1 (8%)	*“Well, he [the participant’s father] doesn’t say it like that, he always says he didn’t have that much time. But I do think so. Yes, I think he also has a bit of a guilty conscience.” [44 years old, survivor, CHEK2]*
3.4. Facing negative reactions from family members	1 (8%)	*“Well, for example, my niece/I have a problem there now. We got along great with each other (...). Therefore, I was so disappointed and hurt. That was really hard.” [50 years old, survivor, CHEK2]*
4. Coping		
4.1. Emotion-focused coping	12 (100%)	*“That you have this awareness of oneself that many things that you don’t want to do, you don’t have to do. Well, this awareness, this mindfulness towards oneself.” [47 years old, survivor, CHEK2]*
4.2. Problem-focused coping	10 (83%)	*“I have this confidence that they’ll find it, and when they do, it’s up to me to also act immediately or to say, okay, right now.” [55 years old, survivor, CHEK2]*
4.3. Meaning-focused coping	5 (42%)	*“And that’s why the breast cancer was a stroke of luck. She [the psycho-oncologist] cleared that up [her anxiety and panic disorder]. I recognized it.” [58 years old, survivor, PALB2]*
5. Impact of familial medical history		
5.1. Intensive experience with death and/or disease in the past	2 (17%)	*“Well, I’m someone, I say of myself, I can handle it pretty well because I’ve seen it all the way to the end happening with my mother.” [38 years old, previvor, CHEK2]*
5.2. Appraisal and evaluation of the way the mother dealt with the disease	5 (42%)	*“My parents have both surrendered to their disease at some point. And I have noticed that, of course. And from my observation, at the moment they surrendered to the disease, it got worse. And I decided for myself right from the start. I won’t do that. I am going to fight that enemy. And I won’t surrender to that enemy.” [49 years old, survivor, PALB2]*
6. Support needs		
6.1. Personal psycho-oncological counseling	3 (25%)	*“Although, I think, I would prefer a one-on-one conversation because sometimes the individual aspects get lost in the group.” [29 years old, previvor, PALB2]*
6.2. Self-help groups	4 (33%)	*“Yes, it would actually be interesting to see how they deal with it. Also, those affected because I just don’t have access to my aunt now who also has it [the pathogenic variant].” [34 years old, previvor, CHEK2]*

BC = breast cancer; total number of women n = 12; percentages shown in the table have been rounded to the nearest whole percent.

**Table 3:** Feelings associated with the disclosure of the genetic test result: Relief.

Relief Because…	Number of Women n (%)	Representative Quote
…I don’t have a *BRCA*1/2 pathogenic variant	3 (35%),¥survivors	*“So for me it was a relief that I don’t have this big gene variation, and in my mind I already saw myself under the knife because of the ovaries.” [57 years old, survivor, CHEK2]*
…I have an explanation and confirmation	1 (8%),¥survivor	*“The goal was actually, maybe I actually wanted to have a confirmation because my aunt got sick.” [47 years old, survivor, CHEK2]*
…I finally have something to hold on to and luckily it is not *BRCA1/2*	1 (8%),¥previvor	*“Because then I finally had something to hold on to. So for me, there were so many people among us who died of it, and no one can tell you why. (...) Yes, it is something. You can explain it now why it was like this for my mother.” [38 years old, previvor, CHEK2]*
….my family members will be included in the multimodal intensified breast surveillance program	1 (8%),¥survivor	*“But the bottom line is I was glad I had that gene because it got my daughter and sister into the trial or into preventive care.” [49 years old, survivor, PALB2]*

Survivor = affected pathogenic variant carrier; previvor = unaffected pathogenic variant carrier; total number of women n = 12; percentages shown in the table have been rounded to the nearest whole percent.

**Table 4:** Feelings associated with the overall situation: Fear and concern.

**Fear and Concern…**	**Number of Women n (%)**	**Representative Quote**
…for the health of children/relatives	2 (17%),¥survivors	*“What worried me a bit was that this was also found in leukemia patients. Then it was like that, my aunt, who is my father’s sister, is now suffering from senile leukemia, and I started to think about whether … [started to cry].” [38 years old, survivor, ATM]*
… about getting BC	2 (17%),¥previvors	*“My mother became ill for the first time at the age of 40. I’m now approaching 40, and of course with every examination more and more panic comes along.” [38 years old, previvor, CHEK2]*
…regarding the BC therapy and its sequelae	1 (8%),¥survivor	*“NO, I say, you’re not going to puncture my port with 500 leukocytes [defined as critical leukopenia]. I found that really/Well, things happen that are really, really bad and then you get scared.” [50 years old, survivor, CHEK2]*
…regarding abnormal findings in the intensified breast surveillance program or a recurrence	1 (8%),¥survivor	*“Yes, of course it is every year, when I drive to Cologne, you are of course super nervous a week before and think, ‘Oh, hopefully it’s not something again.’” [44 years old, survivor, CHEK2]*
…regarding unclear statements and information about the risks associated with the pathogenic variant	2 (17%),¥(previvor, survivor)	*“How high the risk is, because, as I said, of course I was a bit confused now by this statement of my gynecologist. That I was told this 15 percent in Cologne and because of the frequency in the family he said 45 percent now.” [38 years old, previvor, CHEK2]*

BC = breast cancer; survivor = affected pathogenic variant carrier; previvor = unaffected pathogenic variant carrier; total number of women n = 12; percentages shown in the table have been rounded to the nearest whole percent.

**Table 5:** Overview of the different types of coping.

Type of Coping and Number of Women n (%)	Strategies	Number of Women n (%)	Representative Quote
Emotion-focused coping,¥n = 12 (100%)	Family, partner, and friends	8 (67%)	*“Family, friends, that was the most important thing at that time. Well, there is nothing more important.” [50 years old, survivor, CHEK2]*
	Self-care	6 (50%)	*“To do things that are good for me, that makes me feel good and not others, yes. Sometimes you do things when you say, ‘Oh well, yes, come on, I’ll do that now—even though you don’t want to. And you just shouldn’t do that all the time, should you.’” [47 years old, survivor, CHEK2]*
	Psychotherapy and self-help groups	2 (17%)	*“I am so grateful to fate, that she [the psycho-oncologist] built me up.” [58 years old, survivor, PALB2]*
	Sports, nature, and activity	2 (17%)	*“Yes, I always have to deal with it intensely in order to understand everything. When in doubt, go outside. I am a nature person, garden, outside, bike.” [58 years old, survivor, PALB2]*
	Religion	1 (8%)	*“But that [her religious belief] has already given me, has already given me such a foothold again.” [50 years old, survivor, CHEK2]*
	Acceptance and repression	1 (8%)	*“Well, there is not much I can do about my situation anyway.” [47 years old, survivor, CHEK2]*
Problem-focused coping,¥n = 10 (83%)	Participation in the multimodal intensified breast surveillance program	9 (75%)	*“That [the intensified breast surveillance] is the, I think, the be-all and end-all for me. I say that in all honesty.” [55 years old, survivor, CHEK2]*
	Lifestyle changes	4 (33%)	*“Because of course you are feeling better when you have the feeling that you are doing something about it.” [38 years old, previvor, CHEK2]*
	Willingness to undergo (risk-reducing) surgeries	3 (25%)	*“And I say very clearly, if there is something, operate straight away, full stop.” [55 years old, survivor, CHEK2]*
	Awareness of the body, health, and physical changes	3 (25%)	*“Well, I became more sensitive, I already became so because of the disease. So, I watch out more, when does something hurt or I don’t know, and I go see the doctor sooner than I used to.” [44 years old, survivor, CHEK2]*
	Confrontation with the gene and the disease	2 (17%)	*“Look at the enemy, then you’ll know if you can handle it.” [58 years old, survivor, PALB2]*
Meaning-based coping,¥n = 5 (42%)	Opportunity for sharing the BC experience with children, relatives, or other women	3 (25%)	*“But it would also, if she had it, calm me down, because one could hand over one’s own experiences. So, you could also tell them, ‘Listen, even if you do it and you have it, you don’t have to be afraid.’” [47 years old, survivor, CHEK2]*
	Awareness of what is good for oneself and how to enjoy life	2 (25%)	*“You notice that throughout the chemo treatment. You realize what is good for you and what is not good for you. And you become very sensitive thereby.” [47 years old, survivor, CHEK2]*
	Identification and appreciation of personal strengths	2 (17%)	*“The others also said, ‘Man, you got through it really well, you did great. (…) And I was happy and blessed and also proud that I managed it differently. (…) Maybe I even got aware of some personal strengths that I would not have appreciated otherwise.’” [57 years old, survivor, CHEK2]*
	Chance to address pre-existing mental health issues through psycho-oncology	1 (8%)	*“And that’s why the breast cancer was a stroke of luck. She [the psycho-oncologist] cleared that up [her anxiety and panic disorder]. I recognized it.” [58 years old, survivor, PALB2]*

Total number of women n = 12; percentages shown in the table have been rounded to the nearest whole percent.

## Data Availability

Research data are not shared.
